# Radiotherapy for recurrent venous malformation involving the thigh and pelvic organs while preserving organ function: a case report

**DOI:** 10.3389/fcvm.2025.1615584

**Published:** 2026-02-11

**Authors:** Doyoon Kim, Jihoon Maeng, Sunghyun Nam, Hyungsoon Kim, Won Sik Ham, Seo Hee Choi, Jaeho Cho

**Affiliations:** 1 Yonsei University College of Medicine, Seoul, Republic of Korea; 2 Department of Urology, Urological Science Institute, Yonsei University College of Medicine, Seoul, Republic of Korea; 3 Department of Radiation Oncology, Yonsei Cancer Center, Heavy Ion Therapy Research Institute, Yonsei University College of Medicine, Seoul, Republic of Korea

**Keywords:** fertility preservation, intensity-modulated radiotherapy, organ preservation, radiotherapy, venous malformation

## Abstract

**Background:**

Venous malformations (VMs) are congenital vascular anomalies that cause significant pain and dysfunction. Although surgery is the primary treatment, no standardized guidelines exist for extensive or recurrent cases in which surgery is not feasible. Additionally, the role of radiotherapy (RT) in the management of VMs remains unclear.

**Methods:**

A 24-year-old woman with extensive, recurrent VM involving the pelvic cavity, right thigh, and buttocks underwent multiple surgeries and sclerotherapy for over 10 years; however, the disease progressed, infiltrating the pelvic organs, and posed a risk to fertility. The patient presented with severe pain, ureteral obstruction, and menstrual irregularities, all of which significantly affected her quality of life. Considering the high morbidity associated with further surgery, definitive RT was performed. The patient received intensity-modulated radiotherapy (IMRT) at a total dose of 43 Gy in 20 fractions, preceded by ovarian transposition to preserve fertility.

**Results:**

After the treatment, the patient experienced remarkable symptom relief, including pain resolution and improvement in menstrual regularity. Follow-up imaging over five years demonstrated a continued reduction in the lesion size. The menstrual cycles remained regular, and anti-Müllerian hormone levels remained within the normal range.

**Conclusion:**

This case demonstrates the potential of RT as an effective treatment for extensive, recurrent pelvic VMs. RT achieves durable disease control while preserving ovarian function, highlighting its role as a viable alternative in select cases.

## 1 Introduction

Venous malformations (VM) are congenital vascular anomalies characterized by a network of veins with continuous endothelial linings and irregularly distributed vascular smooth muscle cells (1). In approximately 90% of cases, VM occurs sporadically as a unifocal lesion, whereas the remaining 10% present as multifocal lesions associated with genetic mutations (2). Owing to slow blood flow through dilated vessels, blood stagnation can lead to localized intravascular coagulopathy, in which both thrombus formation and thrombolysis actively occur (3).

VMs primarily manifest as skin lesions, often presenting as soft, compressible subcutaneous masses with a light-to-dark blue discoloration that changes upon compression. Unlike arteriovenous malformations, VMs typically lack thrill, bruit, or tenderness. Symptoms usually appear in childhood and progressively worsen, with pain involving the joints and tendons, thrombosis, and location-dependent discomfort, such as migraines and speech difficulties.

VM is primarily diagnosed based on imaging findings. Doppler ultrasonography (US) typically reveals slow or absent blood flow, with a mass appearing hypoechoic, heterogeneous, and compressible (4). On magnetic resonance imaging (MRI), VMs demonstrate intermediate signal intensity on T1-weighted images and hyperintense signals on T2-weighted images (5). Lesions are classified into four grades based on their definition and size: Grade 1 (≤5 cm, well-defined), Grade 2a (>5 cm, well-defined), Grade 2b (≤5 cm, ill-defined), and Grade 3 (>5 cm, ill-defined) (6). Laboratory tests including D-dimer levels may aid in differentiating VMs from other vascular anomalies. Differential diagnosis includes lymphatic malformation, arteriovenous malformation, and pelvic venous insufficiency (7), which may present similarly on imaging but differ in flow dynamics and clinical progression. Doppler ultrasound and contrast-enhanced MRI can assist in distinguishing these entities.

The treatment options for VMs include supportive therapy, sclerotherapy, surgery, and medical therapy with sirolimus. Embolization has also been reported as an effective treatment modality, particularly for well-defined, localized lesions, and is often used in conjunction with sclerotherapy to enhance therapeutic outcomes (7–9). Supportive care focuses on symptom management through compression therapy, pain control with low-dose aspirin or anti-inflammatory drugs, and anticoagulation for intravascular coagulopathy (10). Sclerotherapy, the first-line treatment, is often performed alone or as a preoperative adjunct to reduce the lesion size. The commonly used sclerosing agents include ethanol and bleomycin (11, 12). Surgical excision is reserved for small, well-defined lesions that can be completely removed or for larger lesions with clear margins. Sirolimus has shown promise as an adjunct therapy; however, its optimal treatment duration and long-term safety remain under investigation (13).

Radiotherapy (RT) is a potential treatment option for refractory or extensive VMs, particularly for those that are recurrent or unresponsive to other treatments. However, its use remains limited owing to concerns regarding its effectiveness in benign lesions, potential malignant transformation, and functional impairment. Therefore, the 2017 Japanese Clinical Practice Guidelines for Vascular Anomalies advise caution with RT (14). Nevertheless, emerging evidence suggests that RT may be beneficial in select cases. Ratnayake et al. reported six cases of cavernous VMs in the orbital apex treated with fractionated RT, all of which showed lesion regression and symptom improvement, including reduced proptosis and visual field defects (15). Similarly, Fujita et al. described successful RT management of intra-articular VMs in the knee, whereas Kishi et al. reported a pancreatic arteriovenous malformation that responded favorably to RT (16, 17). These findings indicate that RT could be a viable option for refractory VMs. However, its role in cases involving the visceral organs remains largely unexplored.

This report presents the case of a young unmarried woman with extensive VM primarily involving the right thigh and buttock, which extended into the right pelvic cavity and infiltrated the right side of the vagina, uterus, adnexa, bladder, and pelvic sidewall. The patient was successfully treated with RT, which achieved favorable tumor control while preserving organ function and fertility.

## 2 Case description

**Figure 1:**
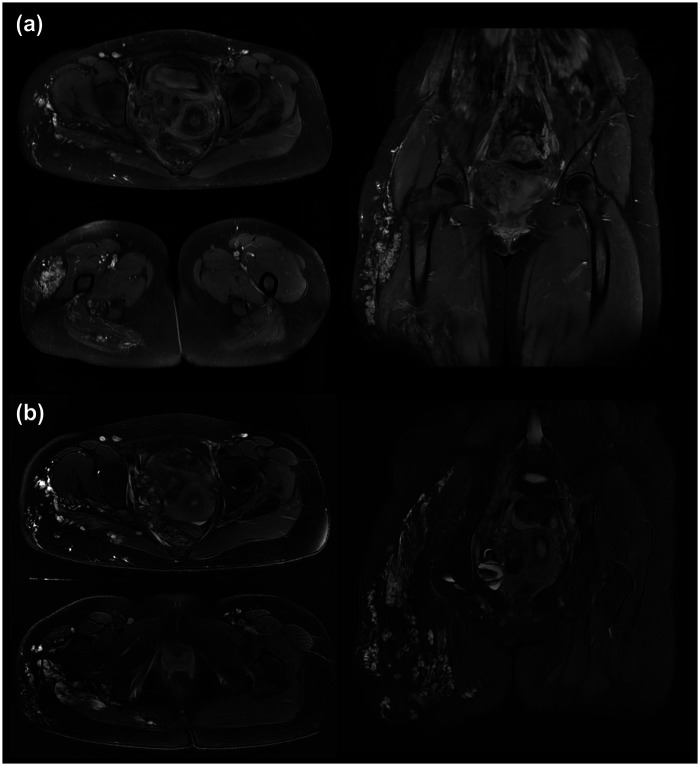
Pre-treatment pelvic MRI images. T1-weighted axial and coronal images **(a)** and T2-weighted axial and coronal images **(b)** from the pretreatment pelvic MRI. The images show a vascular lesion in the right pelvic cavity characterized by ectatic vascular spaces without a definite solid component. The lesion was associated with a right ureteral obstruction, likely due to the mass effect. Multiple scattered vascular malformations were identified in the right thigh and buttocks.

**Figure 2:**
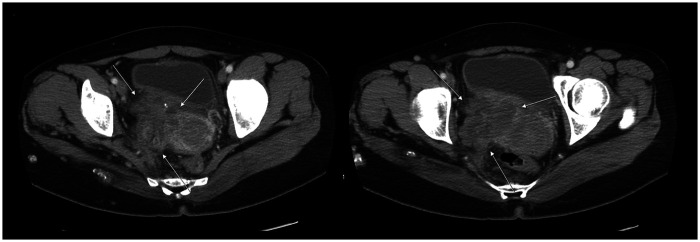
Pre-treatment contrast-enhanced abdominopelvic CT (APCT) images. Pre-treatment APCT demonstrated a 7.5 cm vascular lesion in the right pelvic cavity abutting the right side of the vagina, uterus, adnexa, bladder, and pelvic sidewall and encasing the right internal iliac chain. The lesion was associated with mild right hydronephrosis, likely due to a distal ureteral obstruction. In addition, multifocal enhancing nodular lesions were observed on the right buttocks and thigh.

In June 2018, a 24-year-old female patient presented to the Department of Plastic Surgery at our institution with symptomatic and extensive VM. The patient was initially diagnosed with VM of the right lower extremity at the age of five. Over the course of 19 years prior to visiting our institution, she underwent a total of approximately seven procedural interventions at outside hospitals. These treatments included multiple sessions of surgical excision and sclerotherapy. Despite these repeated interventions, the disease progressed, leading to severe pain and complications including inflammation secondary to right ureteral obstruction. Pelvic MRI (Figure 1) and contrast-enhanced abdominal and pelvic computed tomography (APCT) (Figure 2) revealed a 5 cm mass in the right pelvic cavity invading the right side of the vagina, uterus, adnexa, bladder, and pelvic sidewall, as well as encasing the internal iliac chain. In addition, multiple nodular lesions extending from the right buttock to the thigh were observed. The patient also exhibited multiple nodular lesions in the spleen and liver that were suspected to be hemangiomas.

Multidisciplinary discussion was conducted to determine the most appropriate treatment strategy. Given the extensive invasion of the pelvic mass into the adjacent pelvic organs, surgical resection was deemed unfeasible because of the anticipated need for an extensive procedure with high surgical morbidity and the impracticality of achieving complete excision. Furthermore, considering the patient's young age, the expected deterioration in the quality of life following radical surgery was deemed unacceptable. Therefore, definitive RT was selected as the preferred treatment for this patient.

**Figure 3:**
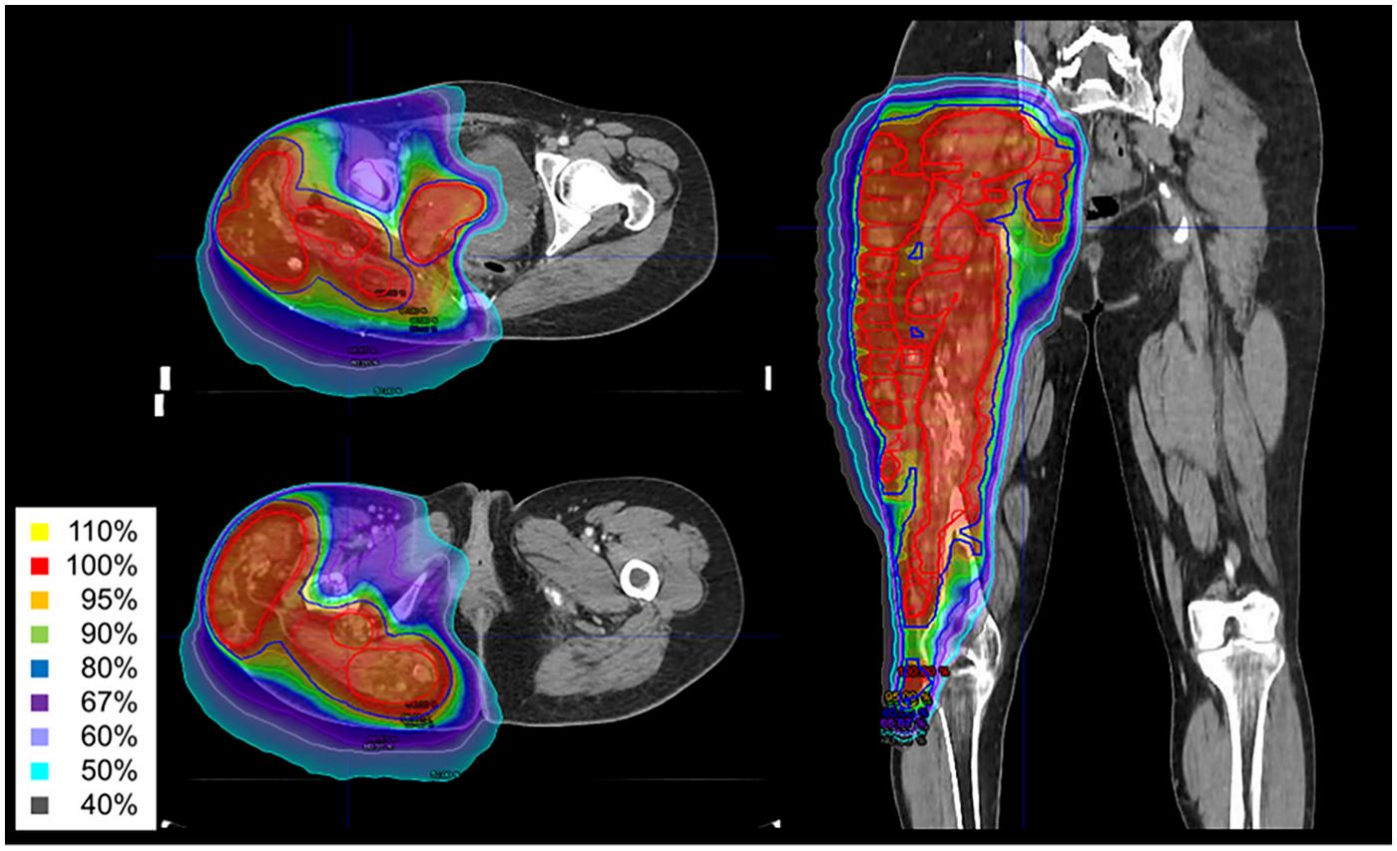
Intensity-modulated radiation therapy (IMRT) plan with isodose line distributions. The red contour represents PTV1, and blue contour represents PTV2. Using the simultaneous integrated boost (SIB) technique, PTV1 and PTV2 received 2.15 Gy and 2.0 Gy per fraction, respectively. The total prescribed dose was 43 Gy delivered in 20 fractions, ensuring effective dose delivery while minimizing radiation exposure to adjacent critical structures.

In the Department of Radiation Oncology, intensity-modulated radiotherapy (IMRT) was planned for the right pelvic and thigh lesions. IMRT was performed using TomoTherapy (Accuray, Inc., Sunnyvale, CA, USA). However, due to the radiation field encompassing the ovaries, there was a high likelihood of radiation-induced ovarian damage. Following a thorough discussion with the patient, ovarian transposition was performed before RT to preserve fertility. The right ovary, left ovary, and right fallopian tube were transposed 2 cm superior to the umbilicus. RT commenced approximately three weeks postoperatively. The planning target volumes (PTV1 and PTV2) were delineated with appropriate margins around the gross lesions. A total dose of 43 Gy was delivered in 20 fractions from July to August 2018, with 2.15 Gy per fraction for PTV1 and 2 Gy per fraction for PTV2 (Figure 3). The maximum dose to the adjacent bowels was 30.85 Gy. The transposed right ovary received a maximum dose of 1.45 Gy and a mean dose of 0.97 Gy, while the left ovary received a maximum dose of 0.31 Gy and a mean dose of 0.26 Gy. The patient completed the planned RT course without any acute adverse effects. Right flank pain, which was present prior to treatment, showed significant improvement at two months post-RT.

**Figure 4:**
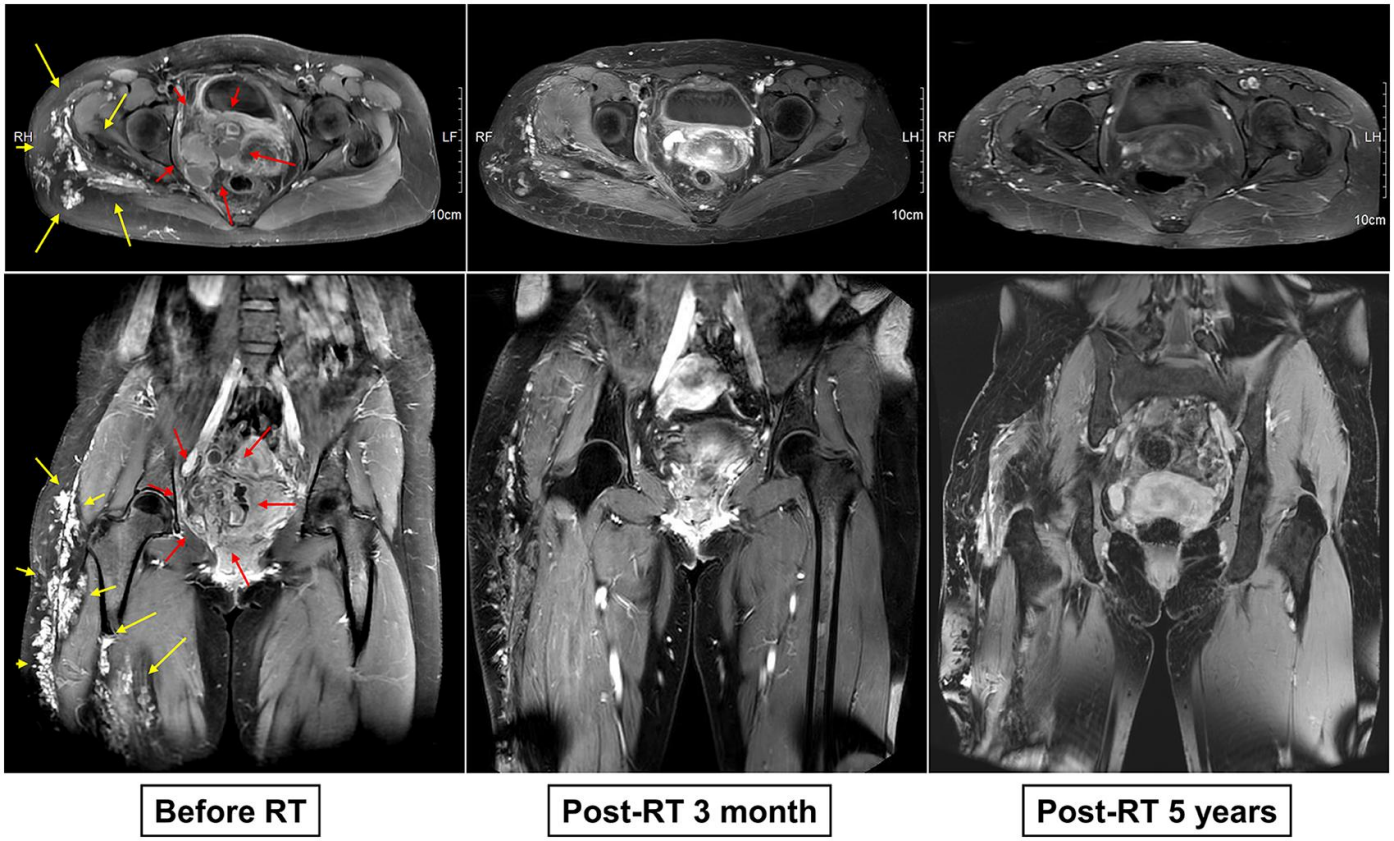
Serial axial and coronal pelvic MRI images before and after radiotherapy. Axial and Coronal MRI scans were obtained at regular intervals before and after radiotherapy (RT), demonstrating a gradual reduction in the vascular lesion size.

The first follow-up MRI, three months after treatment, demonstrated an approximate 30% reduction in the size of the right pelvic mass (Supplementary Figure S1). Subsequent MRI scans, conducted at six-month intervals over a two-year period, indicated a continued gradual reduction in the size of the right pelvic and thigh lesions. At the five-year follow-up (July 2023), MRI confirmed that the treated VM lesions had decreased by approximately 60% compared to the initial size and remained stable with no evidence of recurrence (Figure 4). The patient reported mild discomfort with movement and did not exhibit late RT-related toxicities. Furthermore, the patient, now 31 years of age, maintained regular menstrual cycles throughout the follow-up period without any hormonal abnormalities. The pretreatment serum anti-Müllerian hormone (AMH) level was 2.13 ng/ml. Three months post-RT, AMH levels decreased to 0.59 ng/ml but showed gradual recovery over time, reaching 1.28 ng/ml in July 2024.

## 3 Discussion

VMs are often asymptomatic; however, when lesions are large or located in critical areas, they can cause significant pain and functional impairment. Treatment options include surgery, sclerotherapy, embolization, medical therapy with sirolimus, and supportive care. Surgical resection is generally preferred for localized disease with severe progression, but may be infeasible due to the extent of the disease or contraindications based on patient-specific factors. In such scenarios, embolization—with or without adjunctive sclerotherapy—offers a minimally invasive alternative, particularly for well-defined lesions (7–9). However, its efficacy diminishes in diffuse or infiltrative VMs involving multiple organ systems. For patients unsuitable for surgery or other treatments, RT may serve as a viable option. While RT has traditionally been used for benign conditions such as thyroid ophthalmopathy, heterotopic ossification, and keloids, emerging evidence suggests a potential role in controlling nonmalignant vascular anomalies through inhibition of lesion growth. Several small-scale studies (15–18) have demonstrated the clinical benefits of fractionated RT for VMs at various anatomical locations (Table 1); however, its role remains poorly defined.

**Table 1:** Summary of studies on fractionated radiotherapy for venous malformations.

Author	Year	No. of patients	Disease	RT details	Treatment results
Kishi K et al. (14)	2011	1	Pancreatic arteriovenous malformation	40 Gy (2 Gy/fr)	• Tumor size reduction at 6 months • Lasting effects for 18 months • No significant toxicity
Fujita T et al. (13)	2014	1	Intraarticular venous malformations of the knee	40 Gy (2 Gy/fr)	• Significant shrinkage at 15 months • Reduced pain and swelling • Maintained range of motion
Ratnayake GS et al. (12)	2019	6	Ca vernous venous malformation of the orbital apex	45–50.4 Gy (1.8–2 Gy/fr)	• 63% tumor reduction at 12 months • All lesions decreased in size and remained stable • Symptom improvement in proptosis and visual defects • No treatment-related complications
Liu KX et al. (15)	2021	30	Nonmalignant vascular anomalies (VMs: 5)	Median 39.6 Gy (median 1.9 Gy/fr)	• 47% showed complete or partial response • 90% required additional treatment • Long-term complications included fibrosis, ovarian failure, and radiation pneumonitis • No secondary malignancies

fr, fraction; Gy, gray; RT, radiotherapy; VM, venous malformation.

VMs are often asymptomatic; however, in cases where the lesions are large or located in critical areas, they can cause significant pain and functional impairment. The treatment options include supportive therapy, sclerotherapy, surgery, and medical therapy with sirolimus. Surgical resection is the treatment of choice in patients with severe disease progression. However, no standardized guidelines exist for cases in which surgery is not feasible because of the extent of the disease or contraindicated due to patient-specific factors. Although RT has been utilized for certain benign conditions such as thyroid ophthalmopathy, heterotopic ossification, and keloids, its potential role in nonmalignant vascular anomalies warrants consideration, particularly because of its ability to inhibit rapid lesion growth.

Here, we report the case of a young patient with extensive VM who achieved durable disease remission after IMRT. Despite multiple surgical interventions over a 10-year period, the disease continued to progress, infiltrating critical organs and raising concerns about functional impairment and fertility preservation. Supportive therapy and sclerotherapy were unsuitable because of the extensive nature of the lesion and associated complications, and sirolimus therapy was not available at that time (10, 11, 13). Although medical therapy with sirolimus has recently emerged as a key treatment for complicated VMs, its use for this indication was considered off-label and was not covered by the National Health Insurance in our country at the time of treatment in 2018. Surgical resection was considered impractical due to the high morbidity and complexity of the lesion. RT was selected as a non-invasive and well-tolerated alternative, offering a safe and effective means of managing this challenging case. Using a personalized treatment approach, RT achieved sustained symptom relief and preserved fertility without significant treatment-related toxicity. IMRT was administered at a total dose of 43 Gy to the PTV based on previous studies (15–18), demonstrating that effective tumor control can be achieved with a moderate dose of approximately 40 Gy while minimizing radiation-induced damage to adjacent structures.

VMs can arise due to genetic predispositions or somatic mutations. Inherited somatic mutations associated with venous malformations include TIE2, ALK1, and PTEN, whereas noninherited somatic mutations include TIE2, AKT, PIK3CA, GNAQ, and RAS (19). These mutations are primarily associated with angiogenesis, vascular cell growth, proliferation, and apoptosis (19). Given that VMs exhibit increased cellular growth and proliferation compared with normal tissues, they may be more susceptible to radiation-induced damage. Radiation generates reactive oxygen species (ROS) and induces double-stranded DNA breaks, which are particularly effective against rapidly proliferating cells. When delivered in multiple fractions, RT increases the likelihood of targeting cells in the proliferative phase, thereby enhancing selective damage to venous malformations. Although RT does not result in an immediate therapeutic response (16), previous studies have reported gradual reductions in lesion size (20). Similarly, in our case, the venous malformation demonstrated progressive shrinkage following RT.

Artificial intelligence (AI) has emerged as a valuable tool in clinical medicine, particularly in the interpretation of complex imaging data. In vascular pathologies such as venous malformations, AI-assisted analysis of ultrasound or MRI has demonstrated high diagnostic accuracy, reduced inter-observer variability, and improved detection of subtle abnormalities (21–23). These technologies can also assist in automated lesion segmentation, longitudinal volumetric tracking, and early detection of treatment response or recurrence. Although AI-based image analysis was not utilized in the present case, its incorporation into future clinical workflows may enhance the objectivity and reproducibility of VM evaluation, especially in cases requiring multidisciplinary decision-making or long-term monitoring.

Given the pelvic location of the lesion, the patient was at potential risk of radiation-induced side effects, including gastrointestinal toxicity, infertility, sexual dysfunction, and urinary complications (24–27). Fertility preservation is particularly critical in young female patients undergoing pelvic RT, necessitating a thorough risk-benefit assessment before initiating treatment. If RT is deemed necessary, preemptive interventions should be considered to mitigate its effects on the ovarian function. Premature ovarian failure can significantly affect quality of life, leading to early menopause-related complications. In the present case, ovarian transposition was successfully performed before RT. Moreover, IMRT allowed for effective ovarian sparing, with a mean dose of 0.97 Gy to the right ovary and 0.26 Gy to the left ovary. The patient maintained regular menstrual cycles throughout the follow-up period, and her AMH levels showed partial recovery over time, indicating preserved ovarian function.

In addition to fertility preservation, other potential side effects such as gastrointestinal and urinary tract toxicity should also be considered. The radiation dose administered (40–43 Gy) was lower than the threshold typically associated with severe gastrointestinal or genitourinary toxicity. However, the patient did not experience diarrhea or urinary dysfunction during the four-year post-treatment follow-up period, despite receiving radiation over a relatively large field. In addition, the patient did not report any functional impairment due to muscle contracture. Long-term follow-up remains essential for monitoring late toxicities; however, at the latest follow-up, the patient remained asymptomatic. Notably, she experienced significant pain relief, leading to an improved quality of life without the need for additional medical interventions.

This study has several limitations inherent to its design as a retrospective case report. First, the findings from a single patient cannot be generalized to the broader population of patients with VMs. Second, the absence of a control group limits the ability to definitively attribute the clinical outcome solely to the intervention, although the patient's history of multiple treatment failures serves as a self-control to some extent. Third, while the five-year follow-up demonstrated stable disease control, lifelong monitoring is necessary to fully assess the potential for late radiation-induced toxicities, including secondary malignancies. Therefore, radiotherapy should be considered with caution and is currently recommended only for highly selected patients with extensive, refractory VMs for whom surgical or medical interventions are not feasible.

This case highlights the effectiveness of fractionated RT in treating extensive VMs involving the pelvic cavity, buttocks, and right thigh. Given the limited data available on RT for highly recurrent and extensive VMs, this case provides valuable clinical evidence supporting its potential as an alternative treatment option when surgery is not feasible or causes excessive morbidity. Notably, durable disease control was achieved without significant late toxicities, further emphasizing the clinical relevance of RT in the management of challenging VM cases. These findings suggest that a moderate dose of RT may offer a viable therapeutic approach for refractory VMs, particularly in cases in which conventional treatments have failed. Further research is needed to evaluate its broader applicability and to establish standard guidelines for optimizing RT for the treatment of extensive and recurrent VMs.

## Data Availability

The raw data supporting the conclusions of this article will be made available by the authors, without undue reservation.
