# Supplementary material for: Radiotherapy for recurrent venous malformation involving the thigh and pelvic organs while preserving organ function: a case report
Source: Front Cardiovasc Med. 2026 Feb 11;12:1615584. doi: 10.3389/fcvm.2025.1615584 (PMC12932492; doi:10.3389/fcvm.2025.1615584)

# Supplementary Figure

Supplementary Figure 1. Pelvic MRI images at 3 Months Post-Radiotherapy

The first follow-up pelvic MRI, performed three months after radiotherapy (RT), showed a decrease in the size of the vascular lesions in the right pelvic cavity. However, the diffuse scattered vascular lesions in the right thigh and buttocks remained unchanged.


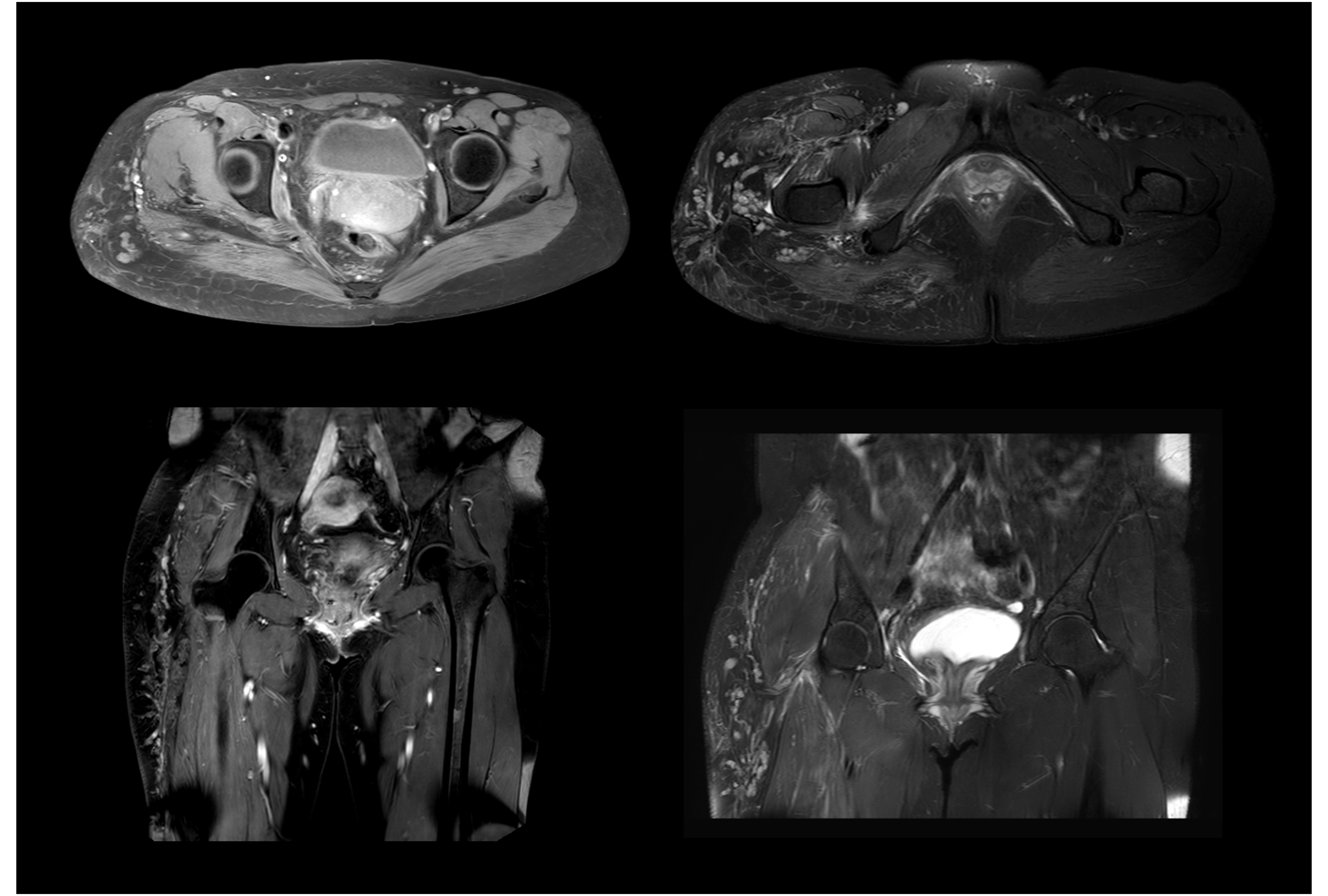

Supplement: Supplementary file 1 [file Datasheet1.docx]
